# Orbitofrontal cortical thinning and aggression in mild traumatic brain injury patients

**DOI:** 10.1002/brb3.581

**Published:** 2016-09-28

**Authors:** Daniel J. Epstein, Margaret Legarreta, Elliot Bueler, Jace King, Erin McGlade, Deborah Yurgelun‐Todd

**Affiliations:** ^1^ Interdepartmental Program in NeuroscienceUniversity of UtahSalt Lake CityUTUSA; ^2^ Cognitive Neuroimaging LaboratorySalt Lake CityUTUSA; ^3^ Salt Lake City Rocky Mountain MIRECCSalt Lake CityUTUSA; ^4^ Department of PsychiatryUniversity of UtahSalt Lake CityUTUSA

**Keywords:** aggression, mild traumatic brain injury, morphometry, orbitofrontal cortex

## Abstract

**Introduction:**

Although mild traumatic brain injury (mTBI) comprises 80% of all TBI, the morphological examination of the orbitofrontal cortex (OFC) in relation to clinical symptoms such as aggression, anxiety and depression in a strictly mTBI sample has never before been performed.

**Objectives:**

The primary objective of the study was to determine if mTBI patients would show morphological differences in the OFC and if the morphology of this region would relate to clinical symptoms.

**Methods:**

Using structural images acquired in a 3T MRI machine, the cortical thickness and cortical volume (corrected for total brain volume) of the OFC was collected for healthy control (*N *= 27) subjects and chronic mTBI (*N *= 55) patients at least one year post injury. Also, during clinical interviews, measures quantifying the severity of clinical symptoms, including aggression, anxiety, and depression, were collected.

**Results:**

MTBI subjects displayed increased aggression, anxiety, and depression, and anxiety and depression measures showed a relationship with the number of mTBI in which the subject lost consciousness. The cortical thickness of the right lateral OFC displayed evidence of thinning in the mTBI group; however, after correction for multiple comparisons, this difference was no longer significant. Clinical measures were not significantly related with OFC morphometry.

**Conclusion:**

This study found increased aggression, anxiety, and depression, in the mTBI group as well as evidence of cortical thinning in the right lateral OFC. The association between clinical symptoms and the number of mTBI with loss of consciousness suggests the number and severity of mTBI may influence clinical symptoms long after injury. Future studies examining other brain regions involved in the production and regulation of affective processes and inclusion of subjects with well‐characterized mood disorders could further elucidate the relationship between mTBI, brain morphology, and clinical symptoms.

## 1 Introduction

The neurobiological basis of aggression has been an active area of research for over a century, and numerous studies have documented a strong relationship between traumatic brain injury (TBI) and aggressive behavior (Damasio, Grabowski, Frank, Galaburda, & Damsio, 1994; Dyer, Bell, McCann, & Rauch, 2006; Harlow, 1848; Kim, 2002; Rao et al., 2009; Ratiu, Talos, Haker, Lieberman, & Everett, 2004; Van Horn et al., 2012). In the United States, the CDC estimates that 1.7 million people experience a TBI annually (Faul, Xu, Wald, & Coronado, 2010). Of these cases, 80%, or 1.3 million, are classified as mild TBI (mTBI); defined by a head injury causing any of the following: disorientation, loss of consciousness for <30 min, or post traumatic amnesia lasting <24 hr (Belanger, Uomoto, & Vanderploeg, 2009; Guerrero, Thurman, & Sniezek, 2000; Kay et al., 1993). This figure likely underestimates the actual number of mTBI suffered every year because it does not include patients that do not seek medical attention, which, when combined, make up an estimated 25% of mTBI cases, according to the CDC (Cassidy et al., 2004; Faul et al., 2010).

Numerous investigations, including studies of prison populations, have examined the association between head injuries and aggressive or criminal behavior. Sarapata, Herrmann, Johnson, and Aycock (1998) observed that 50% of individuals convicted of non‐violent crimes reported suffering a head injury serious enough to require seeking medical care. Slaughter, Fann, and Ehde (2003) expanded on these findings by using standardized assessments to measure aggression in a random sample of incarcerated subjects. In the study, 87% of interviewed subjects reported suffering at least one TBI of some degree, 58% reported having experienced at least one mTBI, and 29% reported suffering a mTBI in the previous year. In addition, subjects who reported having a TBI in the preceding twelve months exhibited increased anger and aggression based on responses to the Brief Anger and Aggression Questionnaire. More recently, a number of studies have shown that TBI is more prevalent in both male and female prisoners than in the general population (Colantionio et al., 2014; Ferguson, Pickelsimer, Corrigan, Bogner, & Wald, 2012; Schofield et al., 2015). TBI is also increased among perpetrators of domestic violence, another indicator that TBI is linked with aggressive behavior (Farrer, Frost, & Hedges, 2012). This association is not just apparent in adults, but in adolescents as well (Farrer, Frost, & Hedges, 2013; Perron & Howard, 2008; Vaughn, Salas‐Wright, DeLisi, & Perron, 2014).

Studies of non‐incarcerated patients reporting head injuries have also displayed significantly increased aggressive behavior (Dyer et al., 2006; Greve et al., 2001). Rao and colleagues divided study participants with varying degrees of TBI severity into those with aggressive tendencies and those without (Rao et al., 2009). They found that subjects in the aggressive TBI group displayed more difficulties performing everyday activities and exhibited reduced social functioning. The investigators suggested that early detection and treatment of aggression could help to diminish these and other negative societal effects.

Neurobiological models of aggression have been proposed to help clarify the basis of these behaviors and are often comprised of brain regions and networks that are conceptualized as either promoting or inhibiting aggressive behavior. Brain regions that are thought to contribute to aggression include the amygdala and insula, while prefrontal areas, including the orbitofrontal cortex (OFC), serve to inhibit aggression (Blair, 2001; Mickzek et al., 2007; Panksepp & Zellner, 2004; Siever, 2008). Damage or metabolic changes in these regions can affect the balance of this initiation/inhibition system and subsequently influence behavior. When TBI is considered using these models, it is likely that damage to the ventral prefrontal cortex disinhibits subcortical generators of aggression (Arciniegas & Wortzel, 2014; Kim, 2002; Pardini et al., 2011; Starkstein & Robinson, 1997). For instance, in a sample of Vietnam War veterans with head injuries, Grafman et al. found that subjects with prefrontal injuries, particularly OFC lesions, displayed increased aggression (Grafman et al., 1996).

The OFC, the most ventral portion of the prefrontal cortex, is structurally and functionally connected to a number of regions including the hippocampus, amygdala, and various cortical areas (Barbas, 2007). The OFC receives gustatory, olfactory and affective input, as well as information regarding decision‐making, and is often divided into medial and lateral regions (Cavada, Company, Tejedor, Cruz‐Rizzolo, & Reinoso‐Suarez, 2000; Ongur & Price, 2000). The medial OFC (MOFC) is an area of convergence; it combines sensory, memory, and reward inputs, while the lateral OFC (LOFC) has an inhibitory role in emotion‐based decision‐making and behaviors (Elliott, Dolan, & Frith, 2000). The LOFC is functionally connected to the dorsolateral prefrontal cortex, an important inhibitory and regulatory region, as well as the amygdala, a critical limbic region that is affectively responsive and may promote aggressive behavior (Kahnt, Chang, Park, Heinzle, & Haynes, 2012). Damage to the OFC can interrupt this affective network, and may lead to an increase in aggression and other clinical symptoms, such as anxiety and depression (Grafman et al., 1996; Kim, 2002; Pardini et al., 2011).

Despite evidence of a relationship between TBI, aggression and the ventral prefrontal cortex, the neurobiological correlates of aggression in mTBI have not been comprehensively explored. An investigation directly assessing the relationship between aggressive behavior and OFC morphometry in subjects who have experienced mTBI has not been reported. Given the findings described above, we hypothesized that the mTBI group would display increased clinical symptoms, including aggression and depression, and decreased OFC cortical thickness and cortical volume. In addition, that changes in OFC morphometry would be associated with clinical symptoms secondary to disinhibition related to OFC integrity.

## 2 Materials and Methods

### 2.1 Subjects

A total of 60 males with at least one mTBI that occurred at least 12 months prior and 27 male healthy control (HC) participants were recruited from the George E. Wahlen Department of Veteran Affairs (VA) Medical Center and the community via local advertisements and by word of mouth. Inclusion criteria for all participants were that subjects be between the ages of 18–55. Exclusionary criteria for all subjects included major sensorimotor handicaps (e.g., deafness, blindness, paralysis), estimated full scale IQ <80, history of claustrophobia, autism, schizophrenia, anorexia nervosa or bulimia, active medical or neurological disease other than TBI that would impact neurobiology or brain function, history of electroconvulsive therapy; and metal fragments or implants that would be contraindicated for MRI. The Institutional Review Boards at the George E. Wahlen VA Medical Center and the University of Utah approved this study. All subjects provided written informed consent prior to participation in this study.

### 2.2 Assessment measures

Participants completed a battery of clinical measures in addition to imaging. The Structured Clinical Interview for DSM‐IV Patient Version (SCID‐I/P) was administered to assess the presence or absence of Axis I disorders (First, Spitzer, Gibbon, & Williams, 2002). Diagnoses were confirmed via clinician consensus. The DSM‐IV‐TR Global Assessment of Functioning (GAF) was used to evaluate global functioning using a scale from 1 (worst) to 100 (best) (American Psychiatric Association, 2000). The Hamilton Anxiety Rating Scale (HAM‐A) and the Hamilton Depression Rating Scale (HAM‐D) were used to measure anxiety and depression symptoms (Hamilton, 1959, 1960). Socioeconomic status was assessed using a modified version of the Hollingshead Four‐Factor Index of Socioeconomic Status (SES) (Cirino et al., 2002). The Buss‐Perry Aggression Questionnaire (BPAQ) was used to assess aggression (Buss & Perry, 1992). The BPAQ is a self‐report survey and is composed of 29 items that are ranked on a 5‐point scale. These items can be broken down into 4 sub‐scores: Physical Aggression, Verbal Aggression, Anger and Hostility, or combined for a BPAQ‐Total score. The HAM scales, the SES, and the BPAQ have been established as being reliable and were administered and scored blindly (Gerevich, Bacskai, & Czobor, 2007; Harris, 1997; Maier, Buller, Philipp, & Heuser, 1988). The clinicians who administered and graded these measures have been verified for inter‐rater reliability (kappa > .90). Group means and standard deviations for these measures can be seen in Table 1.

**Table 1:** Group demographic and clinical data ANCOVA results with age as covariate

	HC (*N *= 27)		mTBI (*N *= 55)		*F*‐Value	Sig.
	Mean	SD	Mean	SD		
Age (One‐way ANOVA)	33.85	8.384	35.75	7.956	0.990	0.323
Buss‐perry aggression questionnaire	57.19	14.107	77.36	20.475	21.818	**<0.001**
Years of education	14.89 (*N *= 27)	2.342	14.13 (*N *= 54)	1.943	2.451	0.121
Socioeconomic status	38.76 (*N *= 25)	13.578	32.15 (*N *= 54)	11.194	4.804	**0.031**
Global assessment of functioning	81.84 (*N *= 25)	9.521	64.52 (*N *= 54)	14.393	28.604	**<0.001**
Hamilton anxiety rating scale	3.154 (*N *= 26)	5.767	11.30 (*N *= 54)	9.432	16.471	**<0.001**
Hamilton depression rating scale	2.231 (*N *= 26)	3.253	9.426 (*N *= 54)	7.573	21.338	**<0.001**

OFC, orbitofrontal cortex. Bold values indicate significant differences before multiple comparisons correction

The Ohio State University‐TBI Identification Method (OSU‐ID) was used to assess the presence, number and severity of lifetime TBI injuries (Corrigan & Bogner, 2007). Participants were classified as having a TBI if they reported an injury to the head followed by an alteration or loss of consciousness (LOC) (Belanger et al., 2009). MTBI was defined as a head injury that resulted in dizziness, confusion, or LOC lasting <30 min and posttraumatic amnesia (PTA) lasting <24 hr, in accordance with the guidelines recommended by the American Congress of Rehabilitation Medicine (Kay et al., 1993). Healthy control participants in the comparison group had no current major DSM‐IV Axis I diagnosis, based on clinical interviews, or history of TBI, based on the Ohio State University‐TBI Identification Method (OSU‐ID). Five mTBI subjects, identified as having additionally experienced at least one moderate or severe TBI, were excluded from the study. Of the remaining 55 mTBI patients, 25 had not experienced any LOC during or after their injuries, while 30 mTBI subjects experienced LOC, lasting <30 min, from at least one head injury. The most severe TBI measure was determined by a trained interviewer (EB) giving the OSU‐TBI and was based on the amount of time that LOC lasted for in addition to the number and severity of post‐concussive symptoms experienced. Mild TBI descriptive statistics included the number of total mTBI, the number of mTBI with LOC, the number of mTBI without LOC, the number of months since the most recent mTBI, and the number of months since the most severe mTBI experienced. Means, standard deviations, and ranges of these measures can be seen in Table 2.

**Table 2:** mTBI descriptive data

	mTBI (*N *= 55)			
	Mean	SD	Min.	Max.
Number of mTBI	5.44	7.036	1	33
Number of mTBI w/o LOC	3.75	6.412	0	32
Number of mTBI with LOC	0.73	0.827	0	3
Time since most recent mTBI (months)	107.29	93.250	12	384
Time since most severe mTBI (months)	149.62	100.50	12	384

### 2.3 Magnetic resonance imaging acquisition

Structural imaging was performed at the Utah Center for Advanced Imaging Research (UCAIR) using a 3T Siemens Trio scanner. This scanner is tested weekly for scan‐rescan reliability. Structural imaging data was acquired using a T1‐weighted 3D MPRAGE GRAPPA sequence acquired in the sagittal plane using a 12‐channel head coil with TE/TR/TI=3.38 ms/2.0s/1.1s, 8° flip, 256 × 256 acquisition matrix, 256 mm^2^ FOV, 160 slices, and 1.0 mm slice thickness. All scans were read by a neuroradiologist to control for gross or focal pathology. No focal or gross pathology was identified for any participant in the current study; therefore no participant was excluded based on these evaluations. The original imaging files were transferred from the scanner in the DICOM format and coded.

### 2.4 Segmentation and morphometric procedure

Brain volume and cortical thickness were calculated using the FreeSurfer software environment (version 4.4), which is free to use and available to download online (http://surfer.nmr.mgh.harvard.edu/). The process used to analyze each subject's brain images is available on the FreeSurfer website. Scans were processed by a blinded technician (JBK). This included motion correction, extraction of brain tissue, transformation to Talairach space, segmentation and parcellation into regions of interest as specified by Deiskan et al. (2006). After processing, scans were inspected by an experienced technician to ensure segmentation and parcellation were performed correctly. Scans were also examined for the presence of artifacts; no artifacts were found. Uncorrected OFC cortical volumes (mm^3^) were divided by the total segmented brain volume (mm^3^) and then multiplied by 1000, which resulted in the corrected cortical volumes. Four regions of interest were investigated: left LOFC, right LOFC, left MOFC, and right MOFC. Each region of interest was studied using two morphometric measures: cortical volume, corrected for total brain volume, and cortical thickness. Means and standard deviations of these measures can be seen in Table 3.

**Table 3:** Group morphometry data and ANCOVA results with age as covariate

	HC (*N *= 27)		mTBI (*N *= 55)		*F*‐value	Sig.
	Mean	SD	Mean	SD		
Left lateral OFC cortical thickness (mm)	2.836	0.139	2.785	0.146	2.253	0.137
Right Lateral OFC cortical thickness	2.874	0.158	2.781	0.133	7.682	**0.007**
Left Medial OFC cortical thickness	2.535	0.191	2.549	0.192	0.022	0.883
Right Medial OFC cortical thickness	2.444	0.171	2.460	0.198	0.104	0.748
Left Lateral OFC cortical volume	5.844	0.572	5.854	0.483	0.072	0.790
Right lateral OFC cortical volume	6.006	0.482	5.856	0.468	1.447	0.233
Left medial OFC cortical volume	3.375	0.496	3.423	0.428	0.282	0.597
Right medial OFC cortical volume	3.537	0.591	3.448	0.450	0.718	0.399

OFC, orbitofrontal cortex. Bold values indicate significant differences before multiple comparisons correction

### 2.5 Statistical analysis

Statistical analyses were carried out using the SPSS software (version 20) for Mac OS X (IBM Corp., 2011). One‐way ANCOVA with age as a covariate was performed with demographic and clinical measures to determine the presence of group differences while correcting for variance in age. One‐way ANCOVA with age as a covariate was also performed with morphometric measures. Within‐group hierarchical regression analyses with age added to the model at the first level were used to evaluate associations between demographic, clinical and structural measures. Bonferroni correction (α = .00625) was used to account for multiple comparisons.

## 3 Results

### 3.1 Group differences in clinical and demographic measures

One‐way ANCOVA analysis was performed with age as a covariate, group as a fixed factor, and demographic and clinical measures as dependent variables, to determine if demographic and clinical measures were different between groups when controlling for age. BPAQ‐Physical, *F*(1,79)  = 29.866, *p* < .001, BPAQ‐Anger *F*(1,79)  = 12.124, *p *= .001, BPAQ‐Hostility *F*(1,79)  = 10.477, *p *= .002, and BPAQ‐Total, *F*(1, 79)  = 21.818, *p *< .001, all showed significant group differences. GAF, *F*(1,76)  = 28.604, *p *= .001, SES, *F*(1, 76)  = 4.804, *p *= .031, HAM‐A, *F*(1, 77)  = 16.471, *p *< .001, and HAM‐D, *F*(1, 77)  = 21.338, *p *< .001, were significantly different between groups as well (Table 1).

### 3.2 Regression analyses for mTBI descriptive data and clinical measures

Hierarchical regression analyses with age included in the first step of the model were used to investigate the relationship between mTBI descriptive measures and both demographic and clinical measures. With BPAQ‐Total as the dependent variable, the number of mTBI with LOC (β = .285, *p *= .035) improved the model with age included (R‐square change = .080, *F*‐change = 4.680, *p *= .035). With GAF as the dependent variable, the number of mTBI with LOC (β = −.347, *p *= .011) improved the model (R‐square change = .119, *F*‐change = 6.960, *p *= .011). With HAM‐A as the dependent variable, the number of mTBI with LOC (β = .385, *p *= .004) significantly improved the model (R‐square change = .147, *F*‐change = 8.893, *p *= .004). With HAM‐D as the dependent variable, the number of mTBI with LOC (β = .414, *p *= .002) significantly improved the model above and beyond the effects of age (F‐square change = .170, *F*‐change = 10.492, *p *= .002). These results can be seen in Table 4.

**Table 4:** Regression between number of mTBI with LOC and clinical measures with age as a covariate

	mTBI (*N *= 55)	
	β	Sig
Buss‐perry aggression questionnaire	.290	.035
Hamilton anxiety rating scale	.386	.004
Hamilton depression rating scale	.412	.002
Global assessment of functioning	−.347	.011
socioeconomic status	−.131	.348

### 3.3 OFC Morphometric group differences

One‐way ANCOVA analysis was performed with age as a covariate, group as a fixed factor, and the cortical thickness and volume of each of the four regions of interest as dependent variables, to determine if OFC regional morphometry differed between groups after controlling for the effects of age. The between‐group difference in right LOFC cortical thickness, *F*(1, 79)  = 7.682, *p *= .007 (Table 3), did not survive Bonferroni correction for multiple comparisons (α = .00625).

### 3.4 Regression analyses for OFC morphometry and demographic and clinical measures

Within‐group hierarchical regression analyses with age included into the model first were performed with the OFC morphometry measures as dependent variables and demographic and clinical measures as independent variables. For the mTBI group, with left LOFC thickness as the dependent variable, BPAQ‐Total (β = −.278, *p *= .042) improved the model (R‐square change = .075, *F*‐change = 4.343, *p *= .042). HAM‐D (β = −.327, *p *= .015) also improved the model with left LOFC thickness as the dependent variable (R‐square change = .107, *F*‐change = 6.276, *p *= .015). With right LOFC as the dependent variable, HAM‐D (β = −.286, *p *= .037) improved the model with age included (R‐square change = .082, *F*‐change = 4.603, *p *= .037). With right MOFC thickness as the dependent variable, SES (β = −.385, *p *= .004) improved the model (R‐square change = .147, *F*‐change 8.857, *p *= .004). For the HC group, with right LOFC thickness as the dependent variable, BPAQ‐Total (β = −.400, *p *= .037) improved the model with age included (R‐square change = .159, F = .4885, *p *= .037). Morphometry regression results did not survive Bonferroni correction for multiple comparisons, except for the relationship between SES and right MOFC thickness.

### 3.5 Regression analyses for OFC morphometry and mTBI descriptive data

With left MOFC as the dependent variable, the number of TBI with LOC (β = .258, *p *= .049) improved the model (R‐square change = .066, *F*‐change = 4.059, *p *= .049); age was also related to left MOFC thickness in the model (β = .322, *p *= .015). With left LOFC volume as the dependent variable, the number of TBI without LOC (β = −.272, *p *= .043) improved the model (R‐Square change = .074, *F*‐change = 4.321, *p *= .043). With right MOFC volume as the dependent variable, the number of months since the most recent mTBI (β = −.445, *p *= .008) improved the model above and beyond the effects of age (R‐square change = .118, *F*‐change = 7.683, *p *= .008); age was also significant in the model (β = .577, *p *= .001). Morphometry regression results did not survive Bonferroni correction for multiple comparisons.

## 4 Discussion

This study compared the cortical volume, adjusted for individual total brain volume, and cortical thickness of the medial and lateral OFC between a group of participants with mTBI and healthy controls. Groups were also compared on a self‐reported measure of aggression, the BPAQ. Lastly, the relationship between OFC morphometry and aggression was explored. It was hypothesized that the structure of the OFC would differ in mTBI subjects relative to healthy controls, and that, overall, aggression and clinical symptoms would be increased in the mTBI population. In addition, it was hypothesized that the morphometry of the OFC would relate to aggression. A reduction in the cortical thickness of the right LOFC was found in the mTBI group, however this finding did not retain statistical significance after correction for multiple comparisons. Self‐reported aggression was increased in the mTBI group and post hoc exploratory analyses also found increased anxiety and depression as well as lower global functioning and SES in these individuals. The postulated relationship between aggression and OFC morphometry was not observed, nevertheless both anxiety and depression symptom severity were associated with the number of mTBI with LOC.

Previous studies using similar imaging and processing methods have found changes in the cortical thickness of the OFC, but to our knowledge this is the first evidence of OFC cortical thinning in a population restricted to mTBI (Kuhn, Schubert, & Gallinat, 2010; Kuperberg et al., 2003; Wilde et al., 2005). MOFC cortical thickness did not differ between groups. This and other research suggests the MOFC is more closely linked to the production of negative affect, while the LOFC has a greater influence on inhibiting actions associated with negative affect, such as those related to aggression, anxiety, and depression (Elliott et al., 2000; Oschner, Bunge, Gross, & Gabrieli, 2002; Phan et al., 2005).

Between‐group differences in cortical thickness were found in the right, but not the left, LOFC. This study hypothesized bilateral decreases, so the lateralized results found in this investigation were unanticipated. The thickness of the right and left LOFC in the mTBI group were roughly equal; the between group difference identified was due to a relatively thicker right LOFC in the HC group. A study by Oschner et al. (2004) found the right LOFC, but not the left, was active during the down regulation of negative emotion, which suggests a lateralization of function within the LOFC. Other investigations have observed an association between left LOFC and aggression, with a study by Gansler et al. (2009). finding the left LOFC was a better predictor of aggression than the right LOFC in psychiatric patients. Another investigation, by Dougherty et al. (2004) found reduced functional activity, using positron emission tomography, in the left ventromedial PFC (VMPFC) in a group of patients with depression and high levels of anger. Taken together, these studies suggest the left and right LOFC perform different functions, however the exact nature of this asymmetry is unclear.

Another noteworthy aspect of this study is that non‐significant between group‐differences were found for the cortical thickness of the right LOFC but not the cortical volume of this region. This could indicate that cortical thickness is a more sensitive measure of injury in mTBI. In part, these results may be due to the fact that cortical thickness metrics were not corrected for total brain volume, which may make cortical thickness a more accurate reflection of mTBI related changes than cortical volume. Past morphometric studies have typically reported either thickness or volume, but not both, which may limit the interpretation of these studies, especially when investigating mTBI. Given the number of investigations that have found significant volumetric changes in TBI samples and the results of this study, it seems warranted that both thickness and volume should be reported when examining the structure of the OFC when studying mTBI (Hudak et al., 2011; MacKenzie et al., 2002; Zhou et al., 2013).

Previous studies have found a relationship between frontal morphometry and aggression in other patient populations. Raine, Lencz, Bihrle, LaCasse, and Colletti (2000) found significant reductions in prefrontal gray matter volume in individuals diagnosed with antisocial personality disorder, a condition strongly associated with aggressive and violent behavior, while Huebner et al. (2008) observed that adolescents with conduct disorder exhibited reduced gray matter volume in a number of areas including the OFC. Aggressive behavior in schizophrenia patients has been shown to correlate with cortical thickness of the OFC, which was also reflected by functional connectivity in this region (Hoptman, Antonius, Mauro, Parker, & Javitt, 2014). However, in this study, associations between a measure of aggression, the BPAQ, and OFC morphometry did not survive correction for multiple comparisons; the findings were therefore not significant. It may be that the morphometry of the OFC is not as strongly linked to aggression in mTBI as it is in other patient groups.

The presence of increased anxiety and depression symptoms in the mTBI group is important to consider. This group reported significantly higher HAM‐A and HAM‐D scores, which quantifies anxiety and depressive symptoms respectively. Further, in the mTBI group a regression model controlling for age, indicated that both HAM‐A and HAM‐D significantly related to BPAQ‐Total. Additionally, HAM‐A and HAM‐D displayed positive relationships with the number of mTBI with LOC, possibly indicating that the accumulation of head injuries may be associated with more severe symptoms. Finally, HAM‐D, but not HAM‐A, displayed non‐significant negative relationships with both left and right LOFC thickness, suggesting a possible connection between OFC morphometry and depressive symptoms. As noted previously, the lateral OFC has been shown to be involved in the inhibition of affective behaviors that originate in the amygdala and other subcortical areas (Elliott et al., 2000; Kahnt et al., 2012). The reduction of OFC cortical thickness could contribute to the exacerbation of aggressive, anxious, and depressive behaviors through disinhibition (Blair, 2001; Mickzek et al., 2007; Panksepp & Zellner, 2004; Siever, 2008).

An active area of discussion within TBI literature is whether TBI causes aggressive behavior or that aggressive people are more likely to sustain a TBI. This topic was investigated in a study carried out by Greve et al. (2001). The authors found that severe trauma survivors who exhibited the greatest amount of aggression had higher levels of pre‐injury aggression. However, multiple studies of felons seem to challenge these findings, as a majority of prisoners self‐report their criminal activity beginning after their first head injury (Colantionio et al., 2014; Ferguson et al., 2012; Sarapata et al., 1998; Schofield et al., 2015; Slaughter et al., 2003). Our study contributes to this area of research, as the mTBI group displayed both increased aggression and a non‐significant finding of cortical thinning in the right LOFC, although aggression and LOFC thickness did not relate directly. This makes it difficult to determine if OFC morphometry is involved in the increased aggression displayed in mTBI. Nevertheless the findings in this paper are demonstrative of a need for further research of aggression, and other clinical symptoms, such as anxiety and depression, in mTBI populations particularly as to how neurobiological mechanisms may relate to these symptoms.

### 4.1 Limitations and Future Directions

Although the TBI group in this study was comprised exclusively of mTBI patients, the possibility remains that even within the mTBI categorization there are degrees of severity that were not captured that could explain the observed variation in aggression, cortical thinning, or both. The most commonly used measure of TBI severity, the Glasgow Coma Scale (GCS), has been used to assess the relationship between TBI severity and behavioral and neurocognitive changes, but the GCS is more useful when the full spectrum of TBI severity is investigated (Gale, Baxter, Roundy, & Johnson, 2005; McCullagh, Oucherlony, Protzner, Blair, & Feinstein, 2001). The OSU‐TBI was used to identity the presence of mTBI in this study. Although this assessment is standardized, has been confirmed to be reliable, and has been used in previous TBI studies, the OSU‐TBI is a self‐reported measure and therefore a possible limitation (Depue et al., 2014; Ferguson et al., 2012). Future studies should attempt to obtain medical records documenting the occurrence of TBI; although this may be more difficult in mTBI populations as a large number of patients do not report to the hospital after a mTBI (Cassidy et al., 2004; Faul et al., 2010). Because of the high prevalence of mTBI, a new metric for measuring severity that is restricted, and more sensitive to mTBI may need to be developed. Future studies should endeavor to quantify the severity of mTBI above and beyond basic classification, if at all possible.

In the present study, anxiety and depression were not matched between groups, and both were related to aggression and OFC morphometry to some degree. Future studies should also consider the role of anxiety and depression when studying aggression in mTBI (Liu et al., 2014; Tu et al., 2012; Van Tol et al., 2010). Studies comparing subjects with mTBI alone, a mood disorder alone, or the combination of the two, would be able to examine the relationship between anxiety and depression, aggression, and TBI more extensively. Studies looking to explicitly study aggression in mTBI should attempt to match for depression and anxiety.

Another limitation of the study was that the control group and mTBI group were not matched for socioeconomic status or substance abuse. The need to do so is supported by the finding of a relationship between right MOFC thickness and SES. However, in regards to both socioeconomic status and substance abuse, the directionality of causation when interacting with TBI is unclear (Bjork & Grant, 2009; Felde, Westermeyer, & Thuras, 2006; Hoofien, Vakil, Gilboa, Donovick, & Barak, 2002; Nordstrom, Edin, Lindstrom, & Nordstrom, 2013; Rassovsky et al., 2015). Further, the groups were not matched for aggressive behavior; a future study comparing aggression‐matched controls would be valuable to more completely evaluate the relationship between OFC morphometry and aggressive behavior in subjects with and without mTBI. An additional limitation was that subjects were not tested for scan‐rescan reliability, only being scanned once.

This study was cross‐sectional and relied on self‐reporting, so it is important not to draw definitive conclusions regarding this issue until longitudinal studies that measure aggression, and other clinical symptoms, preceding and following head injuries are performed. Longitudinal studies would allow for the examination of directionality in the relationship between OFC morphometry and aggression in mTBI, which cannot be determined in the present study.

In addition to longitudinal studies, it would be valuable to explore data from other imaging modalities, and the association with aggression in mTBI. For example, diffusion tensor imaging and water bound pool fraction, which are indirect measures of white matter integrity, could be used to evaluate the relationship between white matter structure with clinical symptoms such as aggression, anxiety and depression (Jorge et al., 2012; Morey et al., 2013; Stikov et al., 2011; Underhill, Rostomily, Mikheev, Yuan, & Yarnykh, 2011). The uncinate fasciculus should be an area of interest because this white matter bundle facilitates neuronal connections to and from the OFC (Von Der Heide, Skipper, Klobusicky, & Olson, 2013). Magnetic resonance spectroscopy could also be used to study between group differences in neurotransmitters and metabolites in the OFC and associated regions (Cady et al., 1996; Kierans et al., 2014; Sivak et al., 2014). Another modality that would be useful is functional connectivity, which measures the correlation of neural activity in different brain regions (Nathan et al., 2015; Raichle et al., 2001; Sours et al., 2015). Using resting state functional connectivity, the impact of the OFC on aggression could be studied by observing changes in functional connectivity between the OFC and functionally and structurally connected regions such as the amygdala, the dorsolateral prefrontal cortex, the thalamus, the striatum, and primary sensory areas (Barbas, 2007; Cavada et al., 2000; Elliott et al., 2000; Bonelli & Cummings, 2007).

## 5 Conclusion

In this study we found reduced right LOFC cortical thickness in a sample of mTBI subjects, which did not survive correction for multiple comparisons. We also found that mTBI subjects exhibited increased aggression, as measured by the Buss‐Perry Aggression Questionnaire. Further, we found increased anxiety and depression and reduced global functioning and SES in the mTBI group. Post hoc analyses confirm that anxiety and depression symptom severity increased as the number of mild TBI involving the loss of consciousness increased, suggesting a relationship between mTBI and anxiety and depression. Non‐significant relationships between aggression and right LOFC morphometry in the HC group and left LOFC morphometry in the mTBI group were observed, and non‐significant relationships between depression symptom severity and bilateral LOFC morphometry were also found in the mTBI group. This is the first reported finding of OFC thinning in a mTBI population. Further, it corroborates previous findings of increased aggression, anxiety, and depression after mTBI and suggests that the OFC may play a role in the modulation of behaviors related to clinical symptoms after mTBI.

## Conflict of Interests

No competing financial interests exist.
